# Time course of nitric oxide synthases, nitrosative stress, and poly(ADP ribosylation) in an ovine sepsis model

**DOI:** 10.1186/cc9097

**Published:** 2010-07-05

**Authors:** Matthias Lange, Rhykka Connelly, Daniel L Traber, Atsumori Hamahata, Yoshimitsu Nakano, Aimalohi Esechie, Collette Jonkam, Sanna von Borzyskowski, Lillian D Traber, Frank C Schmalstieg, David N Herndon, Perenlei Enkhbaatar

**Affiliations:** 1 Department of Anesthesiology, Investigational Intensive Care Unit, The University of Texas Medical Branch and Shriners Burns Hospital for Children, 301 University Boulevard, Galveston, Texas 77550, USA; 2 Department of Anesthesiology and Intensive Care, University of Muenster, Albert-Schweitzer-Str. 33, 48149 Muenster, Germany; 3 Department of Plastic and Reconstructive Surgery, Tokyo Women's Medical University, 8-1 Kawada-cho Shinjuku-ku, Tokyo 162-8666, Japan; 4 Department of Pediatrics, Investigational Intensive Care Unit, The University of Texas Medical Branch and Shriners Burns Hospital for Children, 301 University Boulevard, Galveston, Texas 77550, USA; 5 Department of Surgery, Investigational Intensive Care Unit, The University of Texas Medical Branch and Shriners Burns Hospital for Children, 301 University Boulevard, Galveston, Texas 77550, USA

## Abstract

**Introduction:**

Different isoforms of nitric oxide synthases (NOS) and determinants of oxidative/nitrosative stress play important roles in the pathophysiology of pulmonary dysfunction induced by acute lung injury (ALI) and sepsis. However, the time changes of these pathogenic factors are largely undetermined.

**Methods:**

Twenty-four chronically instrumented sheep were subjected to inhalation of 48 breaths of cotton smoke and instillation of live Pseudomonas aeruginosa into both lungs and were euthanized at 4, 8, 12, 18, and 24 hours post-injury. Additional sheep received sham injury and were euthanized after 24 hrs (control). All animals were mechanically ventilated and fluid resuscitated. Lung tissue was obtained at the respective time points for the measurement of neuronal, endothelial, and inducible NOS (nNOS, eNOS, iNOS) mRNA and their protein expression, calcium-dependent and -independent NOS activity, 3-nitrotyrosine (3-NT), and poly(ADP-ribose) (PAR) protein expression.

**Results:**

The injury induced severe pulmonary dysfunction as indicated by a progressive decline in oxygenation index and concomitant increase in pulmonary shunt fraction. These changes were associated with an early and transient increase in eNOS and an early and profound increase in iNOS expression, while expression of nNOS remained unchanged. Both 3-NT, a marker of protein nitration, and PAR, an indicator of DNA damage, increased early but only transiently.

**Conclusions:**

Identification of the time course of the described pathogenetic factors provides important additional information on the pulmonary response to ALI and sepsis in the ovine model. This information may be crucial for future studies, especially when considering the timing of novel treatment strategies including selective inhibition of NOS isoforms, modulation of peroxynitrite, and PARP.

## Introduction

Severe sepsis and septic shock continue to be major causes of morbidity and mortality of ICU patients [1]. Among the sources of nosocomial infections, ICU-acquired pneumonia represents the leading cause of death [2,3]; and *Pseudomonas *was the second most frequently identified bacteria species causing sepsis among ICU patients in a recent multi-center, observational study [4].

Previous studies revealed the important roles of the different isoforms of nitric oxide (NO) synthases (NOS), peroxynitrite (ONOO^-^), and poly-ADP ribose (PAR) in the pathophysiology of cardiopulmonary derangements induced by acute lung injury (ALI) and sepsis, thereby offering potentially new treatment options such as inhibition of NOS [5], decomposition catalyzation of ONOO^- ^[6], or inhibition of PAR polymerase (PARP) [7].

When considering possible treatment strategies of patients with sepsis, however, it may be crucial to identify the time changes of the expression of the above mentioned pathogenic factors. The present study was therefore conducted to determine the time course of endothelial NOS (eNOS), neuronal NOS (nNOS), inducible NOS (iNOS), 3-nitrotyrosine (3-NT), an index of protein nitration and ONOO^-^, as well as PAR in lung tissue using an established ovine model of sepsis induced by ALI and instillation of live *Pseudomonas *bacteria into the lungs [8].

## Materials and methods

This study was approved by the Animal Care and Use Committee of the University of Texas Medical Branch and conducted in compliance with the guidelines of the National Institutes of Health and the American Physiological Society for the care and use of laboratory animals.

### Animal model

The ovine model of ALI and sepsis induced by smoke inhalation and instillation of *Pseudomonas aeruginosa *into the lungs has been previously described in detail [8,9]. In brief, 24 adult female sheep (body weight, expressed in means ± standard error of the mean (SEM), 34 ± 1 kg) were surgically prepared for chronic study with a femoral artery catheter, a pulmonary artery thermodilution catheter, and a left atrial catheter. After a recovery period of five to seven days, the animals received tracheostomy followed by inhalation injury with 48 breaths of cotton smoke (< 40°C) using a modified bee smoker. Afterward, a stock solution of live *P. aeruginosa *(2-5 × 10^11 ^colony-forming units, from a burn patient at Brooke Army Medical Center; San Antonio, TX, USA) suspended in 30 mL of 0.9% saline solution was instilled into the right middle and lower lobe and left lower lobe of the lung (10 mL each). Anesthesia was then discontinued, and the sheep were allowed to awaken.

### Experimental protocol

The animals were randomly allocated to be euthanized 4, 8, 12, 18, and 24 hours after the injury, respectively (n = 4 per time point). Four additional sheep received sham injury and were euthanized after 24 hours to serve as the uninjured control group. All sheep were mechanically ventilated (Servo Ventilator 900C, Siemens; Elema, Sweden) with a tidal volume of 12 to 15 mL·kg^-1 ^and a positive end expiratory pressure of 5 cmH_2_O. Notably, sheep require higher tidal volumes than humans because the ovine lung compliance is higher and the ovine dead space/tidal volume ratio is larger. The fraction of inspired oxygen (FiO_2_) was set at 1.0 for the first three hours post-injury and was then adjusted to maintain sufficient oxygenation (arterial oxygen saturation (SaO_2_) > 95%, partial pressure of arterial oxygen (PaO_2_) > 90 mmHg) whenever possible. The respiratory rate was initially set at 20 breaths·min^-1 ^and was then adjusted to maintain the partial pressure of arterial carbon dioxide (PaCO_2_) within 5 mmHg of the baseline value. All animals were fluid resuscitated, initially started with an infusion rate of 2 mL·kg^-1 ^h^-1 ^lactated Ringer's solution and adjusted to maintain hematocrit (± 3) and cardiac filling pressures at baseline values. During the study period, all animals had free access to food, but not water. After completion of the experiment, the animals were deeply anesthetized with ketamine and xylazine and euthanized by intravenous injection of saturated potassium chloride. Immediately after death, the lower lobe of the right lung was removed. The bacterial infection spots were detected by gross appearance. Avoiding these spots, a 1 cm-thick section was excised for molecular biological measurements [8].

### Pulmonary hemodynamics, oxygenation and shunting

Arterial and venous pressures were measured from the femoral and pulmonary artery catheters using pressure transducers (model PX3X3, Baxter Edwards Critical Care Division, Irvine, CA, USA) which were connected to a hemodynamic monitor (model 7830A, Hewlett Packard; Santa Clara, CA, USA). Cardiac output (CO) was measured by the thermodilution technique using a CO computer (COM-1, Baxter Edwards Critical Care Division, Irvine, CA, USA). Blood gases were measured using a blood gas analyzer (Synthesis 15, Instrumentation Laboratories; Lexington, MA, USA). Pulmonary vascular resistance, shunt fraction (Qs/Qt), and oxygenation index (PaO_2_/FiO_2_) were calculated using standard equations.

### Immunoblotting in lung tissue homogenates

NOS, 3-NT, PAR, p65, and IL-8 protein expressions were measured using a western blot protocol as described previously [5]. Blots were quantified by NIH IMAGE J scanning densitometry, and normalized to total actin expression.

### Measurement of nitric oxide synthases mRNA in lung tissue homogenates (RT-PCR)

Total RNA was obtained using a commercially available total RNA purification kit (Purescript; Gentra Systems, Inc., Minneapolis, MN, USA). Quantitative PCR of NOS was performed as described previously [8]. The copy numbers were normalized between samples using glyceraldehyde 3-phosphate dehydrogenase (GADPH) copy numbers determined with an external standard constructed from the *v-erb *gene. All results were expressed as copy numbers per microgram of total RNA.

### Measurement of nitric oxide synthase activity in lung tissue homogenates

NOS activity was evaluated by conversion of L-[3H]arginine to L-[3H]citrulline with a NOS activity assay kit according to the manufacturer's instructions (Cayman Chemical, Ann Arbor, MI, USA).

### Measurement of plasma nitrate/nitrite levels

The NO levels were evaluated by measuring the plasma concentration of the intermediate and end products, nitrate/nitrite (NOx), as described previously [10]. For conversion of nitrate to nitrite, the plasma samples were mixed with vanadium (III) and hydrochloric acid at 90°C in the NOx reduction assembly (Antek model 745, Antek Instruments, Houston, TX, USA). Thereafter, the NO reacted with ozone in the reaction chamber of the chemiluminescent NO detector (Antek model 7020, Antek Instruments, Houston, TX, USA), and the emitted light signal was recorded by dedicated software as the NOx content (μmol/L).

### Statistical analysis

All values are expressed as means ± standard error of the mean (SEM). The statistical analysis was performed using the one-way analysis of variance followed by a *post hoc *Dunnett's test as the multiple comparison method. A value of *P *< 0.05 was regarded as statistically significant.

## Results

### Systemic hemodynamics, metabolism, and inflammation

The double hit injury induced a hypotensive-hyperdynamic circulation and significant decreases in both arterial pH and base excess. A systemic inflammatory response was evidenced by a temporary increase in body core temperature and a progressive decrease in white blood cell counts in injured sheep (Table 1).

**Table 1:** Time changes in systemic hemodynamics, metabolism, and inflammation

		Time after injury (hours)				
		
	Control	4	8	12	18	24
MAP, mmHg	105 ± 1	108 ± 10	87 ± 4^a^	81 ± 4^a^	76 ± 11^b^	63 ± 2^c^
CO, L/min	3.8 ± 0.2	5.4 ± 0.4	5.4 ± 0.1	6.6 ± 0.6^a^	6.9 ± 0.5^a^	7.1 ± 0.3^b^
apH, -log_10 _[H^+^]	7.50 ± 0.02	7.60 ± 0.03	7.50 ± 0.07	7.51 ± 0.02	7.37 ± 0.04^a^	7.29 ± 0.06^a^
aBE, mmol/L	2.1 ± 0.8	3.9 ± 1.9	-0.4 ± 2.0	0.3 ± 1.3	-3.2 ± 1.8	-3.6 ± 2.0^a^
PaCO_2_, mmHg	31 ± 0.0	30 ± 2	32 ± 4	30 ± 1	34 ± 2	38 ± 3^a^
BCT, °C	39.6 ± 0.1	40.7 ± 0.7	41.0 ± 0.2	40.6 ± 0.1^a^	40.1 ± 0.4^a^	39.2 ± 0.5
WBC, K/μL	6.7 ± 1.4	3.2 ± 1.0^a^	1.8 ± 0.6^b^	2.2 ± 0.6^b^	1.8 ± 0.5^b^	1.1 ± 0.2^c^

Values recorded before sacrifice of animals with sham injury (control) and at different time points after induction of sepsis following acute lung injury. Each group includes four animals.

aBE, arterial base excess; apH, arterial pH; BCT, body core temperature; CO, cardiac output; MAP, mean arterial pressure; PaCO_2_, partial arterial carbon dioxide pressure; WBC, white blood cells. ^a ^*P *< 0.05, ^b ^*P *< 0.01, ^c ^*P *< 0.001 vs. control group.

### Pulmonary hemodynamics, ventilatory pressures, oxygenation, and shunting

**Figure 1:**
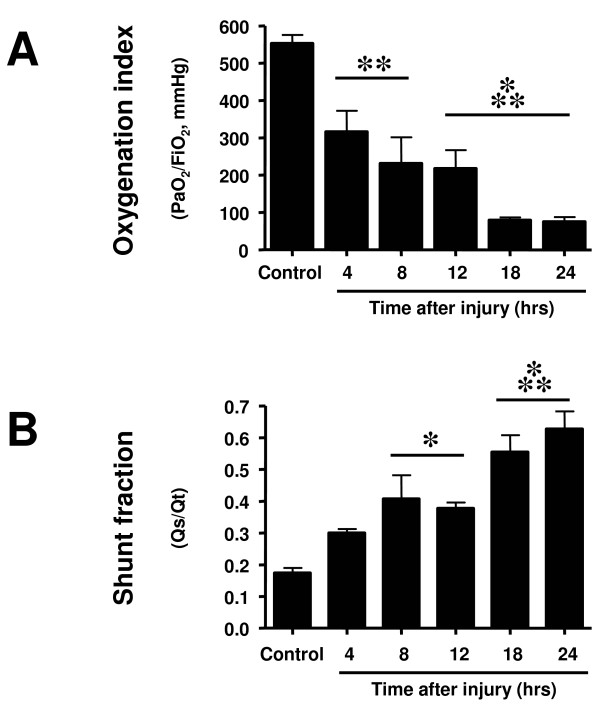
**Time changes in (a) pulmonary oxygenation index and (b) pulmonary shunt fraction**. Measurements were taken before the sacrifice of animals with sham injury (control) and at different time points after induction of sepsis following acute lung injury. FiO_2_, fraction of inspired oxygen; PaO_2_, partial pressure of arterial oxygen. * *P *< 0.05, ** *P *< 0.01, *** *P *< 0.001 vs. control group.

The injury was associated with an early deterioration of pulmonary oxygenation as indicated by a progressive decline in PaO_2_/FiO_2 _ratio. This index was decreased below 200 mmHg at 18 hours after the injury, indicating acute respiratory distress syndrome. The impairment of oxygenation was associated with a concomitant increase in pulmonary shunt fraction (Figure 1). Pulmonary hemodynamics remained stable after the injury, except for significant increases in pulmonary capillary wedge pressure at 12 and 24 hours post-injury. Ventilatory pressures significantly increased over time (Table 2).

**Table 2:** Time changes in pulmonary hemodynamics and ventilatory pressures

		Time after injury (hours)				
		
	Control	4	8	12	18	24
MPAP, mmHg	24 ± 2	24 ± 2	28 ± 3	26 ± 2	31 ± 3	28 ± 2
PVR, mmHg	186 ± 25	134 ± 24	156 ± 22	150 ± 14	180 ± 29	145 ± 11
PCWP, mmHg	12 ± 1	15 ± 1	18 ± 1^a^	14 ± 1	16 ± 1	19 ± 2^b^
Ppeak, cmH_2_O	20 ± 1	21 ± 2	24 ± 4	22 ± 3	30 ± 2^a^	31 ± 2^a^
Ppause, cmH_2_O	16 ± 1	19 ± 2	22 ± 3	20 ± 3	25 ± 2^a^	25 ± 3^a^

Values recorded before sacrifice of animals with sham injury (control) and at different time points after induction of sepsis following acute lung injury. Each group includes four animals.

MPAP, mean pulmonary arterial pressure; PCWP, pulmonary capillary wedge pressure; Ppeak, peak airway pressure; Ppause, pause airway pressure; PVR, pulmonary vascular resistance. ^a ^*P *< 0.05, ^b ^*P *< 0.01.

### Time course of nitric oxide synthases mRNA and protein expressions in lung tissue

**Figure 2:**
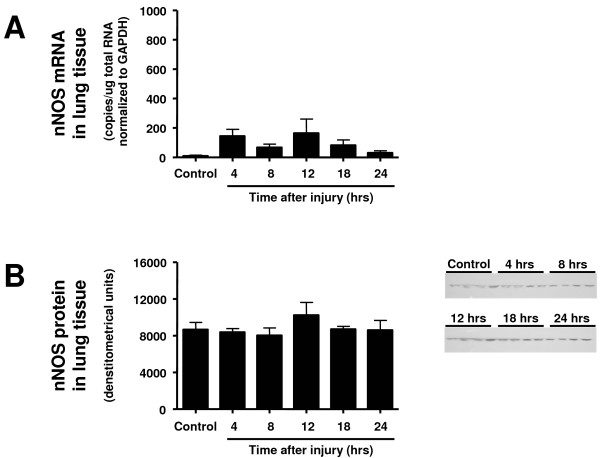
**Time course of (a) neuronal nitric oxide synthase (nNOS) mRNA determined by RT-PCR and (b) nNOS protein expression determined by western blotting in lung tissue at different time points after induction of sepsis following acute lung injury**. Animals with sham injury served as controls.

**Figure 3:**
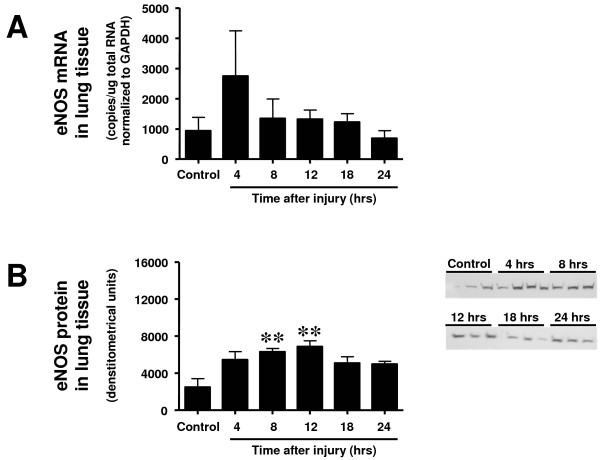
**Time course of (a) endothelial nitric oxide synthase (eNOS) mRNA determined by RT-PCR and (b) eNOS protein expression determined by western blotting in lung tissue at different time points after induction of sepsis following acute lung injury**. Animals with sham injury served as control group. ** *P *< 0.01 vs. control group.

**Figure 4:**
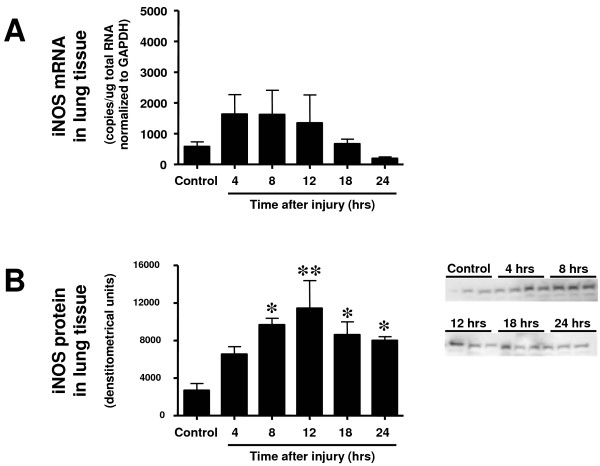
**Time course of (a) incucible nitric oxide synthase (iNOS) mRNA determined by RT-PCR and (b) iNOS protein expression determined by western blotting in lung tissue at different time points after induction of sepsis following acute lung injury**. Animals with sham injury served as control group. * *P *< 0.05, ** *P *< 0.01 vs. control group.

Neither the expression of nNOS protein nor mRNA in lung tissue was increased toward the sham-injured control group at any investigated time point (Figure 2). Expression of eNOS protein was significantly increased at 8 and 12 hours after the injury (Figure 3b) and iNOS protein expression was found significantly increased from 8 to 24 hours (Figure 4b). Although there were no statistically significant increases in mRNA at any time point, eNOS mRNA tended to be increased compared with the control group at 4 hours and iNOS mRNA from 4 to 12 hours post-injury (*P *> 0.05; Figures 3a and 4a).

### Time course of nitric oxide synthase activity in lung tissue and plasma nitrite/nitrate levels

**Figure 5:**
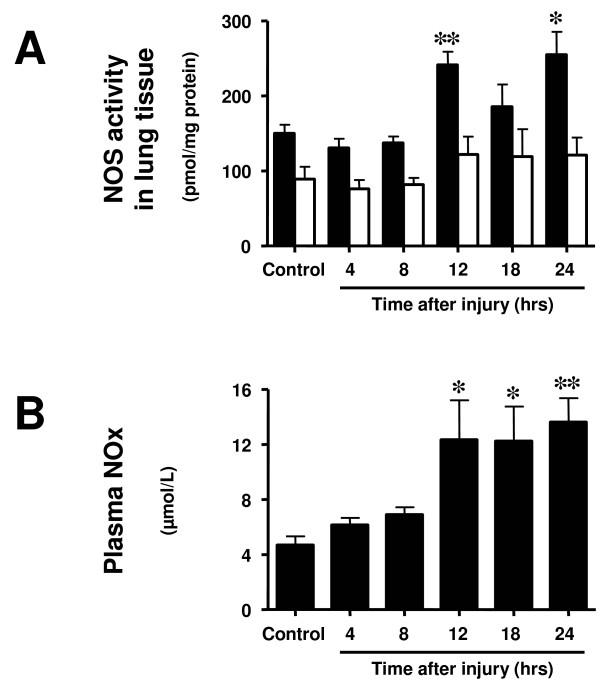
**Time course of (a) calcium-dependent nitric oxide synthase (NOS) activity (total NOS activity, black bars) and calcium-independent NOS activity (inducible NOS activity, open bars) measured in lung tissue and (b) plasma nitrite/nitrate (NOx) levels at different time points after induction of sepsis following acute lung injury**. Animals with sham injury served as control group. * *P *< 0.05, ** *P *< 0.01 vs. control group.

Calcium-dependent NOS (total NOS) activity was significantly increased at 12 and 24 hours after the injury, whereas calcium-independent (iNOS) activity only tended to be higher than in the control group from 12 to 24 hours (*P *> 0.05; Figure 5a). Plasma NOx levels were found significantly increased from 12 to 24 hours after the injury (Figure 5b).

### Time course of 3-nitrotyrosine, poly(ADP ribose), p65, and interleukin-8 protein expression in lung tissue

**Figure 6:**
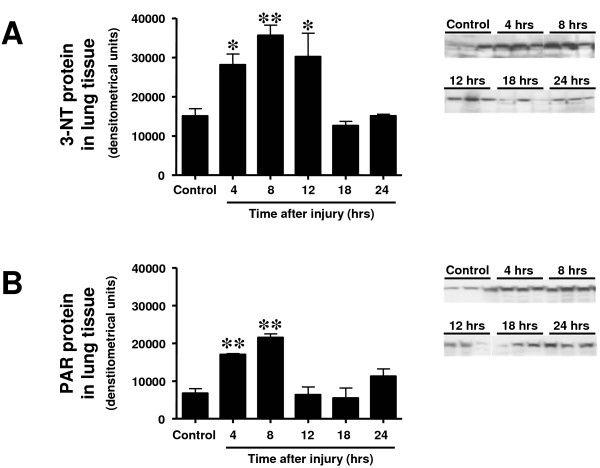
**Time course of (a) 3-nitrotyrosine (3-NT) and (b) poly(ADP ribose) (PAR) protein expression determined by western blotting in lung tissue at different time points after induction of sepsis following acute lung injury**. Animals with sham injury served as control group. * *P *< 0.05, ** *P *< 0.01 vs. control group.

**Figure 7:**
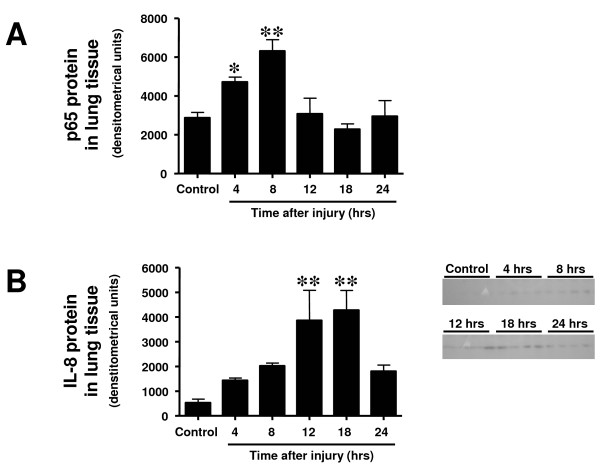
**Time course of (a) p65 and (b) IL-8 protein expression determined by western blotting in lung tissue at different time points after induction of sepsis following acute lung injury**. Animals with sham injury served as control group. * *P *< 0.05, ** *P *< 0.01 vs. control group.

3-NT protein, a marker of protein nitration and ONOO^-^, was increasingly expressed from 4 to 12 hours post-injury. Both expression of PAR and p65 protein was significantly increased at four and eight hours as compared with the control group (Figures 6 and 7). IL-8 protein was increasingly expressed 12 and 18 hours after the injury (Figure 7).

## Discussion

In the present study, induction of sepsis following ALI contributed to an early and severe deterioration of pulmonary function, which was associated with early over-expression of eNOS and iNOS, enhanced NOS activity, and increased expression of markers of nitrosative stress and DNA damage in lung tissue.

The pulmonary response to ALI and sepsis in sheep has been comprehensively studied in previous experiments [5,8,9]. It has been demonstrated that excessively produced NO may exert cytotoxic effects by reacting with superoxide radicals from activated neutrophils, thereby yielding reactive oxygen and nitrogen species such as ONOO^-^. ONOO^- ^in turn may induce cell damage by oxidizing and nitrating/nitrosating proteins and lipids [11,12]. Furthermore, ONOO^- ^can induce excessive activation of the nuclear repair enzyme PARP [13,14], which may cause ATP depletion and cell damage [14,15]. Together, these changes can induce endothelial damage, pulmonary capillary hyperpermeability, and pulmonary edema [9], resulting in severe deterioration of the pulmonary gas exchange.

Increased knowledge of these pathomechanisms provides novel therapeutical options for patients with ALI and sepsis such as inhibition of NOS [5,16] and PARP [7] or decomposition catalyzation of ONOO^- ^[6]. In this regard, extensive research has been conducted to identify the roles of the three different isoforms of NOS. It is commonly believed that NO produced by constitutively expressed isoforms (nNOS and eNOS) is implicated in important physiologic processes, whereas excessively produced NO by iNOS is suspected to be critically involved in the pathophysiology of various diseases including sepsis and ALI [17,18]. Increasing evidence suggests that not only is iNOS-derived NO, in part, responsible for the cardiopulmonary derangements following ALI or sepsis, but so is NO from constitutively expressed nNOS and eNOS [19-22]. In this regard, the results from previous studies suggested beneficial effects of selective NOS inhibition in ALI and sepsis [23-25] at the time of their maximum activity. In contrast, non-selective inhibition of NOS [16] or selective inhibition of different NOS isoforms at the wrong time points may be ineffective or even detrimental [10,26]. Likewise, inhibition of PARP in septic sheep only partially attenuated the sepsis-related cardiopulmonary derangements [7]. The wrong timing of interventions may provide an explanation for these failures in treatment, and thus examination of the pulmonary tissue response at different time points may deliver valuable information for treatment strategies in future experiments.

Excessive NO production may not only be attributed to over-expression of NOS, but also to enhanced activity of constitutively expressed enzymes. Therefore, to profoundly understand the roles of different NOS isoforms in ALI and sepsis, we measured mRNA, protein expression, and enzyme activity in lung tissue, as well as plasma levels of stable NO metabolites in the present study. Although neither mRNA nor protein expression of nNOS was increased at any of the evaluated time points, both eNOS and iNOS protein expressions started to increase early after the injury. Albeit the changes in mRNA of eNOS and iNOS were not statistically significant, they tended to be elevated prior to the increase in protein expression of the respective isoenzyme. Subsequent to enhanced transcription and expression of eNOS and iNOS, both total NOS activity and plasma NOS levels were increased from 12 to 24 hours after the double-hit injury. Interestingly, total NOS, but not iNOS, activity was significantly increased by the injury, suggesting that constitutively expressed NOS contributed substantially to the increases in NOS activity. With the applied methods, it cannot be differentiated between nNOS and eNOS activity. It is therefore conceivable that both isoforms were involved.

Increased expression of 3-NT, a marker of inflammation-related processes and ONOO^-^, and PAR, an index of DNA damage, in lung tissue were early events after the injury, and protein expression returned to values of control animals at 18 and 12 hours, respectively. This was possibly due to decreased ONOO^- ^and PARP activity in the later course of the injury or simply to the fact that a majority of cells with high ONOO^- ^production and PARP activation had already died. The latter assumption is supported by the early peak of p65 protein expression, a subunit of the nuclear factor-kappaB, in lung tissue. Regardless, it is obvious that pharmacologic intervention such as ONOO^- ^decomposition catalyzation or PARP inhibition starting later than 12 hours after injury must become less effective in this model. On the other hand, if pharmacologic interventions have started early, more cells may be vital at later time points and thus treatment may still be efficient later than 12 hours post-injury.

The up-regulations of NOS, ONOO^-^, and PAR protein were followed by a transient increase in protein contents of the pro-inflammatory IL-8 in the lung. For technical reasons, it was not feasible to measure the plasma concentrations of inflammatory cytokines in sheep, but it is conceivable that the increase in IL-8 protein in the lung was secondary to an elevation in blood concentrations.

The current study design does not allow detection of causative mechanisms. It can be discussed, however, that the deterioration in pulmonary function was probably not solely due to the excessive increase in plasma NOx concentrations caused by increased iNOS expression, because these were later events and the pulmonary oxygenation index was already markedly reduced as early as four hours after the injury. More likely, the earlier occurring up-regulation of eNOS may have contributed to increased ONOO^- ^production and PARP activation which, in turn, may have induced endothelial cell damage in the lung. For this purpose, small amounts of eNOS-derived NO appear to be enough, because plasma NOx levels were not increased at this early time point. Alternatively, the increase in constitutive NOS-derived NO may have been missed due to the absence of data on lung tissue NOS activity at earlier time points than four hours. When studying Figure 1, however, it becomes apparent that both oxygenation index and shunt fraction markedly worsened between 12 and 18 hours post-injury. It can be speculated that these secondary deteriorations were now due to the up-regulation of iNOS and excessively produced NO, which increased pulmonary shunting phenomena thereby further impairing pulmonary oxygenation.

There are some limitations of the study we want to acknowledge. First, the study was designed to monitor the sepsis-related pulmonary tissue response for 24 hours after the injury and, unfortunately, we were not able to include more time points for tissue harvesting in this large animal model. It is thus conceivable that we missed the respective time point of peak protein expression and/or activity of nNOS. In this context, experimental evidence revealed that increased activity and expression of nNOS may occur earlier than four hours in the paraventricular nucleus of rats subjected to lipopolysaccharide injection [27]. Second, it may be unexpected that eNOS expression was increased in the present study because eNOS is supposed to be a constitutive enzyme, which cannot be increasingly expressed. However, it needs to be considered that the present investigation evaluated protein expressions and enzyme activities in whole lung homogenates, but not in single cells. In this regard, previous studies demonstrated that constitutive NOS can be expressed by circulating cells, such as neutrophils [28,29]. Consequently, an injury-related increase in inflammatory cells may account for increased protein expression of constitutive NOS in the current study. The discussed issues may be addressed in future studies utilizing genetically modified mice (e.g. nNOS or eNOS deficient). This approach may allow for elimination of possible interactions between NOS isoforms and inclusion of numerous time points due to reduced costs of a small animal model. It further needs to be regarded as a limitation of the current study that the time changes in some parameters may have missed statistical significance due to the relatively low number of animals per group. In addition, the present study investigated female subjects only, and thus gender-specific differences in time changes of NOS, 3-NT, and PARP could not be detected. In this context, it has previously been reported that inhibition of PARP showed protective effects only in male rodents subjected to ischemic stroke or endotoxin-induced inflammation [30-32]. Female gender *per se *provided protection against these injuries. However, pharmacologic inhibition of PARP also had protective effects in female subjects of different species [33].

## Conclusions

The current study describes the time course of NOS isoform expression and NOS activity as well as important markers of ONOO^- ^and PARP activation in lung tissue of sheep subjected to ALI and sepsis. This detailed information may greatly enhance the understanding of pathophysiologic alterations in our ovine model. The identification of the time changes of the described pathogenetic factors may ameliorate the timing of treatment strategies in future studies.

## Key messages

• The development of early and severe pulmonary dysfunction following inhalation injury and pneumonia in sheep was associated with early over-expression of eNOS and iNOS but not nNOS protein in the lung.

• These changes were further associated with enhanced NOS activity and increased expression of markers of nitrosative stress and DNA damage in lung tissue.

• The identification of the time changes of the described pathogenetic factors may ameliorate the timing of treatment strategies in future studies.

## Abbreviations

3-NT: 3-nitrotyrosine; ALI: acute lung injury; CO: cardiac output; eNOS: endothelial nitric oxide synthase; FiO_2_: fraction of inspired oxygen; GAPDH: glyceraldehyde 3-phosphate dehydrogenase; IL: interleukin; iNOS: inducible nitric oxide synthase; nNOS: neuronal nitric oxide synthase; NO: nitric oxide; NOS: nitric oxide synthase; NOx: nitrate/nitrite; ONOO^-^: peroxynitrite; PaCO_2_: partial arterial carbon dioxide pressure; PaO_2_: partial arterial oxygen pressure; PAR: poly(ADP ribose); PARP: poly-ADP ribose polymerase; PCR: polymerase chain reaction; RT-PCR: reverse transcription polymerase chain reaction; Qs/Qt: pulmonary shunt fraction; SEM:0 standard error of the mean.

## Competing interests

The authors declare that they have no competing interests.

## Authors' contributions

PE, DLT, and ML were responsible for the study design and drafted the manuscript. RC and AE performed the immunoblots and helped with the interpretation of the results. AH, YN, CJ, and AE carried out the experiments, participated in the design of the study and helped with the interpretation of the results. LDT performed the complicated surgeries and critically revised the manuscript for important intellectual content. FCS supervised the RT-PCR and helped with the interpretation of the data. DNH revised the manuscript for important intellectual content. SvB contributed to the statistical analysis and interpretation of the data. All authors read and approved the final manuscript.
